# First Insights into the Venom Composition of Two Ecuadorian Coral Snakes

**DOI:** 10.3390/ijms232314686

**Published:** 2022-11-24

**Authors:** Josselin A. Hernández-Altamirano, David Salazar-Valenzuela, Evencio J. Medina-Villamizar, Diego R. Quirola, Ketan Patel, Sakthivel Vaiyapuri, Bruno Lomonte, José R. Almeida

**Affiliations:** 1 Biomolecules Discovery Group, Universidad Regional Amazónica Ikiam, Km 8 Via Muyuna, Tena 150101, Ecuador; 2 Centro de Investigación de la Biodiversidad y Cambio Climático (BioCamb) e Ingeniería en Biodiversidad y Recursos Genéticos, Facultad de Ciencias de Medio Ambiente, Universidad Indoamérica, Quito 180103, Ecuador; 3 School of Biological Sciences, University of Reading, Reading RG6 6UB, UK; 4 School of Pharmacy, University of Reading, Reading RG6 6UB, UK; 5 Instituto Clodomiro Picado, Facultad de Microbiología, Universidad de Costa Rica, San Jose 11501, Costa Rica

**Keywords:** coral snake, Ecuador, mass spectrometry, *Micrurus*, phospholipase A_2_, three-finger toxins, venomics

## Abstract

*Micrurus* is a medically relevant genus of venomous snakes composed of 85 species. Bites caused by coral snakes are rare, but they are usually associated with very severe and life-threatening clinical manifestations. Ecuador is a highly biodiverse country with a complex natural environment, which is home to approximately 20% of identified *Micrurus* species. Additionally, it is on the list of Latin American countries with the highest number of snakebites. However, there is no local antivenom available against the Ecuadorian snake venoms, and the biochemistry of these venoms has been poorly explored. Only a limited number of samples collected in the country from the Viperidae family were recently characterised. Therefore, this study addressed the compositional patterns of two coral snake venoms from Ecuador, *M. helleri* and *M. mipartitus*, using venomics strategies, integrating sample fractionation, gel electrophoresis, and mass spectrometry. Chromatographic and electrophoretic profiles of these snake venoms revealed interspecific variability, which was ascertained by mass spectrometry. The two venoms followed the recently recognised dichotomic toxin expression trends displayed by *Micrurus* species: *M. helleri* venom contains a high proportion (72%) of phospholipase A_2_, whereas *M. mipartitus* venom is dominated by three-finger toxins (63%). A few additional protein families were also detected in these venoms. Overall, these results provide the first comprehensive views on the composition of two Ecuadorian coral snake venoms and expand the knowledge of *Micrurus* venom phenotypes. These findings open novel perspectives to further research the functional aspects of these biological cocktails of PLA_2_s and 3FTxs and stress the need for the preclinical evaluation of the currently used antivenoms for therapeutic purposes in Ecuador.

## 1. Introduction

Venoms are multi-component systems with the ability to recognise cellular and molecular targets, resulting in a complex set of alterations in tissue architecture and function that are associated with long-term complications and/or vital consequences [1]. The variable combination and abundance of proteins and peptides in these mixtures play a determining role in the mechanism of action and pattern of destabilising effects caused by the venoms [2,3]. The venom proteome variability has an impact on the toxin-neutralising capabilities of traditional and modern antivenoms [4,5]. Additionally, venom composition studies constitute a valuable platform for an improved understanding of evolutionary mechanisms [6] and their potential in drug discovery [7,8].

The development of omics technologies has shaped toxinology studies, opening and building novel frontiers and areas, now recognised as venomics, anti-venomics and toxico-venomics [9,10]. These emerging and integrative fields provide a detailed view of the biomolecular richness, functionality, and immunogenicity of venoms [11]. The data obtained by using these strategies support many steps in the antivenom development, from the selection of the immunogenic pool to the evaluation of the cross-reactivity [12]. Specifically, venomics is a proteomic-based technology useful in the early stage of the rational toxin-targeted antivenom [13]. The current application of this approach has shed light on the biochemical inventory of more than 132 snake venoms [14]. However, the catalogue and relative abundance of toxins from specimens of some low-income countries affected by snakebites, such as Ecuador, remains largely unexplored.

Ecuador is a South American nation characterised by the largest number of reptile species per unit area [15]. Its diverse ecosystems are home to around 240 species of snakes, 36 of which are classified as venomous. They are mostly distributed in two families, Elapidae (19) and Viperidae (17), which are responsible for a high number of bites. Proteomics workflows have unravelled the main venom components of a few species of the Viperidae family in Ecuador [16,17,18,19]. However, to the best of our knowledge, Ecuadorian coral (*Micrurus*) venom proteomes have not been analysed previously.

*Micrurus* is a wide-ranging genus, found from the southern United States to northern Argentina [20]. The investigation of coral venoms has been mainly hampered by venom availability [21]. Only 20% of *Micrurus* venoms worldwide have been analysed with proteomics approaches [22] due to the small quantities of venom that we can obtain from these specimens and the difficulties of their adjustment to captive conditions [20]. Curiously, these previous studies have pointed out that *Micrurus* venoms contain a variable amount of low-molecular weight toxins, predominantly phospholipase A_2_ (PLA_2_) or three-finger toxins (3FTxs) [23].

**Figure 1:**
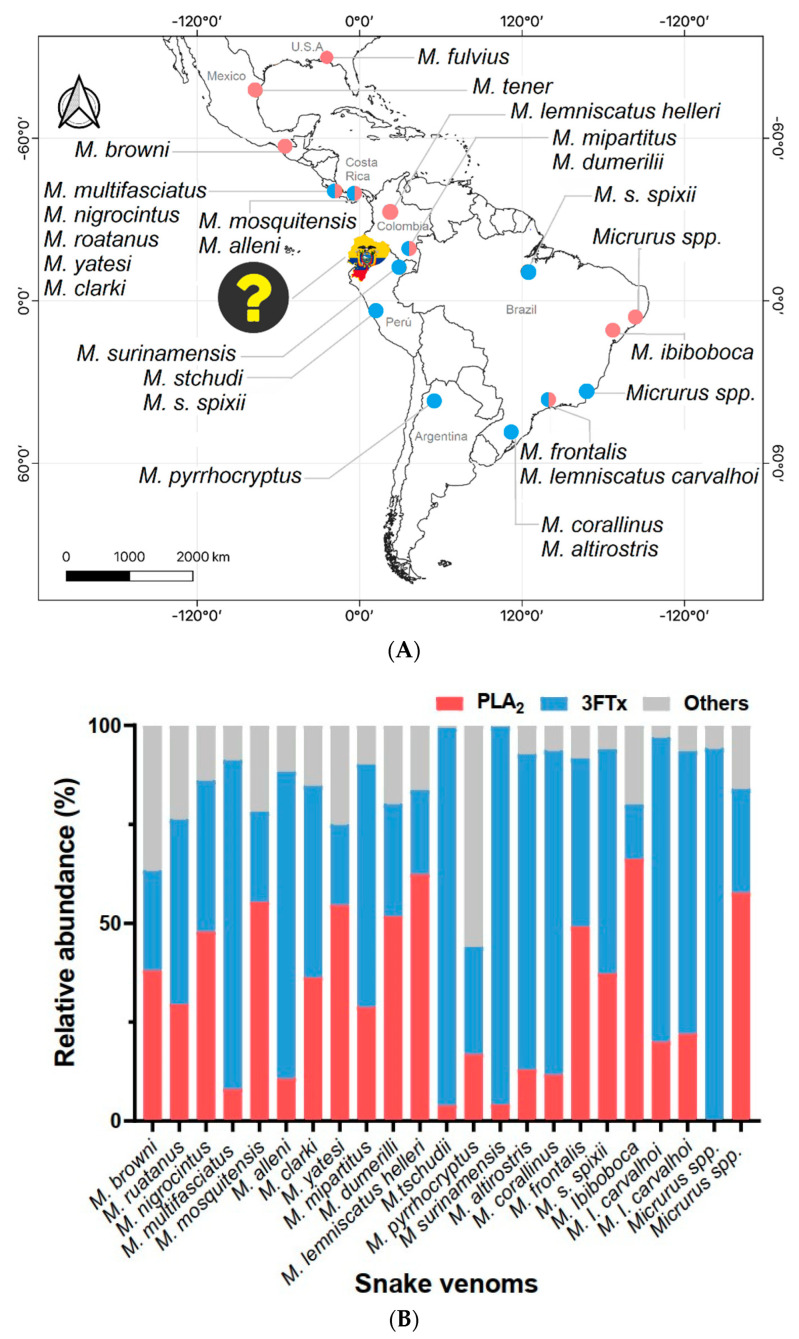
The current knowledge about *Micrurus* venom in the literature. (**A**) *Micrurus* specimens collected from different countries such as Argentina, Brazil, Colombia, Costa Rica, Mexico, and United States were proteomically characterised (dots represent geographical sites where analysed venoms/specimens were collected). However, the composition of Ecuadorian coral venom is still unknown. The question mark (yellow) points out this gap in the understanding of coral snake venoms in a biodiverse region. Red dots represent specimens with a PLA_2_-predominant profile, while blue dots highlight venoms with a higher abundance of 3FTXs. Dual-colour dots illustrate regions where both compositional patterns have been identified. (**B**) Detailed profile of key venom toxins in Elapid snake venoms. PLA_2_-dominant or 3FTx-dominant profiles dictate an intriguing general trend in the distribution of proteins in venoms along the American continent.

Many of the venom characterisation studies involving coral snakes have used specimens from countries with local antivenom production for Elapid snakes, such as Costa Rica [24], Brazil [25], Colombia [26], and Mexico [27]. Figure 1A outlines the geographical origins from where the major *Micrurus* species were used for proteomics research. A remarkable dichotomy in venom phenotypes of *Micrurus* venoms (Figure 1B) has been identified on a north–south axis along the American continent [28]. Several authors agree that more venomics analyses will provide a more realistic picture of this trend [29,30]. These distinct patterns may have relevant translational implications for the snakebite therapy [20,23,29]. The manufacturing of horse-derived antivenom has been discontinued in Ecuador since 2012, and therefore, the country imports polyvalent antivenom produced using *Bothrops asper*, *Crotalus durissus*, and *Lachesis muta* to treat Viperidae accidents [31,32]. On the other hand, Elapidae envenomations are a rare but represent a significant health problem due to the lack of or scarce availability of anti-elapid antivenoms in the public healthcare system [33]. In this context, the present study, for the first time, compared the venom proteomes of two coral snake species collected in Ecuador and provides insights into the challenging puzzle of coral venom composition across the New World.

## 2. Results

### 2.1. Collection of Venoms and Their Fractionation

**Figure 2:**
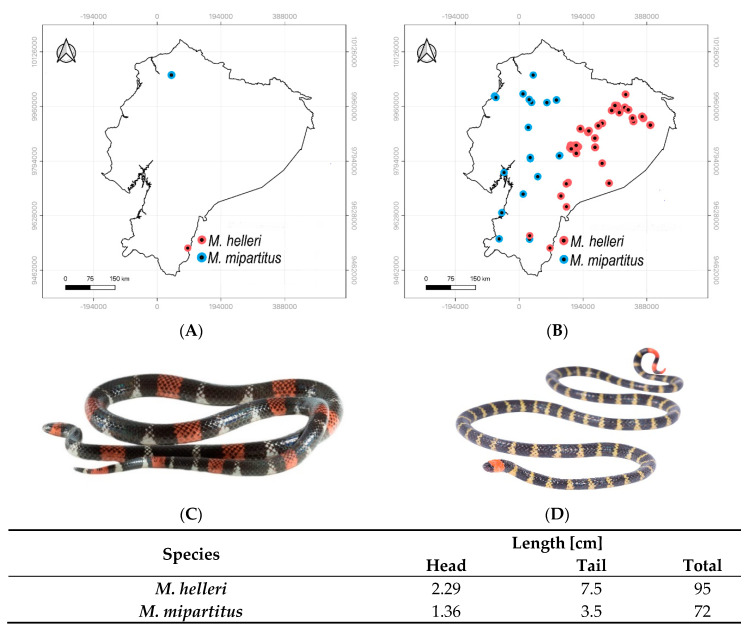
Venom collection and species occurrences of Ecuadorian coral snakes. (**A**) Sample from *M. helleri* (red) was extracted from a specimen found in Nangaritza (Zamora Chinchipe), while venom from *M. mipartitus* (blue) was obtained from a snake found in Quinindé, Jevon Forest Biological Station (Esmeraldas). (**B**) Spatial distribution of these two *Micrurus* species in Ecuador. This map was constructed using geospatial data from (GBIF, http://www.gbif.org) and VertNet (http://www.vertnet.org). Both biodiversity-related repositories were accessed on 18 September 2022. (**C**) *M. helleri* (photo by María José Quiroz; reproduced here from https://bioweb.bio, accessed on 1 November 2022, under a CC BY-NC-ND 4.0 License) and (**D**) *M. mipartitus* specimens (photo by Diego R. Quirola).

Venom samples studied here were extracted from two female snakes found in the wild (Ecuador), one from each species (*M. helleri* and *M. mipartitus*). Figure 2A details the species, localities, and individual characteristics, and Figure 2B shows the occurrences of these two species in Ecuador based on biodiversity-related datasets available in (GBIF, http://www.gbif.org) and VertNet (http://www.vertnet.org) websites (accessed on 1 September 2022).

**Figure 3:**
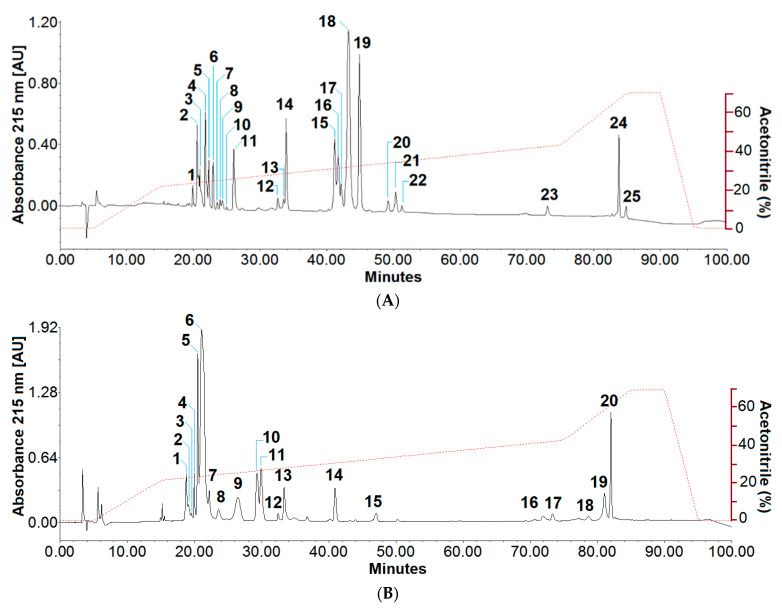
Fractionation of the Ecuadorian coral venoms of (**A**) *M. helleri* and (**B**) *M. mipartitus* by RP-HPLC. The peaks were identified according to their elution through a C18 column for further characterisation by electrophoresis and mass spectrometry analyses. The separation of venom toxins was performed in the gradient (red-dashed lines) elution mode described in the methodology section.

The fractionation of crude venoms by reverse-phase high-performance liquid chromatography revealed different elution profiles, which suggest variability in the combination of toxins. Comparison of the chromatograms showed variations in the number, peak area, and retention time of proteins. In summary, 25 and 20 venom fractions were obtained from *M. helleri* (Figure 3A) and *M. mipartitus* (Figure 3B), respectively. The first venom profile showed highest peaks that emerged between 40 and 50 min, while the biochemical separation of *M. mipartitus* venom presented major fractions approximately at 20 min. According to the absorbance values and peak areas, fraction 18, which eluted at a retention time of 42 min, had the highest relative abundance in *M. helleri*, representing ~34% of the proteome. In the case of *M. mipartitus*, peak 6 had a relative abundance of ~40% and eluted at approximately 20 min.

### 2.2. Biochemical Characterisation of Toxins

**Figure 4:**
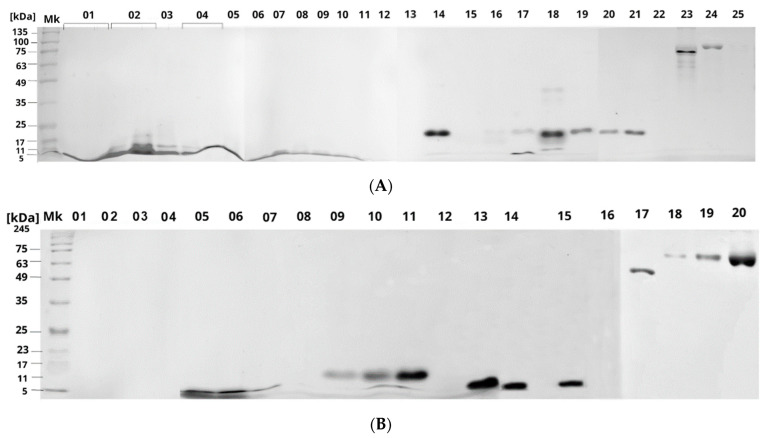
Electrophoretic profiles of Ecuadorian coral snake venom fractions of (**A**) *Micrurus helleri* and (**B**) *Micrurus mipartitus*. Low-molecular mass proteins (5–17 KDa) are highly abundant in these two samples of Ecuadorian elapids. The last chromatographic fractions contain higher molecular mass toxins, around 60 KDa.

RP-HPLC fractions manually collected were subjected to SDS-PAGE under reducing conditions. The two crude venoms showed a clear predominance of low-molecular weight proteins (<20 KDa), mainly between 5 and 17 KDa (Figure 4A,B). Nonetheless, higher molecular weight toxins, mainly of approximately 60 KDa, were observed in the last chromatographic fractions. *M. helleri* venom is characterised by intense bands around 14 KDa (Figure 4A). However, the electrophoretic separation of the venom components from *M. mipartitus* showed a high abundance of bands of lower molecular weight, below 11 KDa (Figure 4B).

### 2.3. Protein Family Assignment by Mass Spectrometry

Bands excised from SDS-PAGE gels and specific chromatography fractions were subjected to proteolytic digestion to identify toxin families using a proteomics approach. The sequences obtained by tandem mass spectrometry were analysed with MASCOT software. Proteins belonging to six families were found in the venom of *M. helleri* (Table 1). The first third of the chromatogram (fractions 2–11) was composed of peptides and 3FTxs. The central region showed the presence of PLA_2_s, which were the most abundant components from this sample. Hyaluronidase and L-amino acid oxidases were detected in the last fractions.

The analysis of tryptic-digested peptides from the chromatographic fractions and electrophoretic bands of *M. mipartitus* venom revealed the presence of four toxins families, with 3FTxs as the most prominent group (Table 2). These low-molecular mass proteins were mainly identified in the first fractions, although some of them were also observed in the central region of the chromatogram, such as fractions 13–15. The last collected toxins correspond to snake venom metalloproteases (SVMPs) and LAOs.

**Figure 5:**
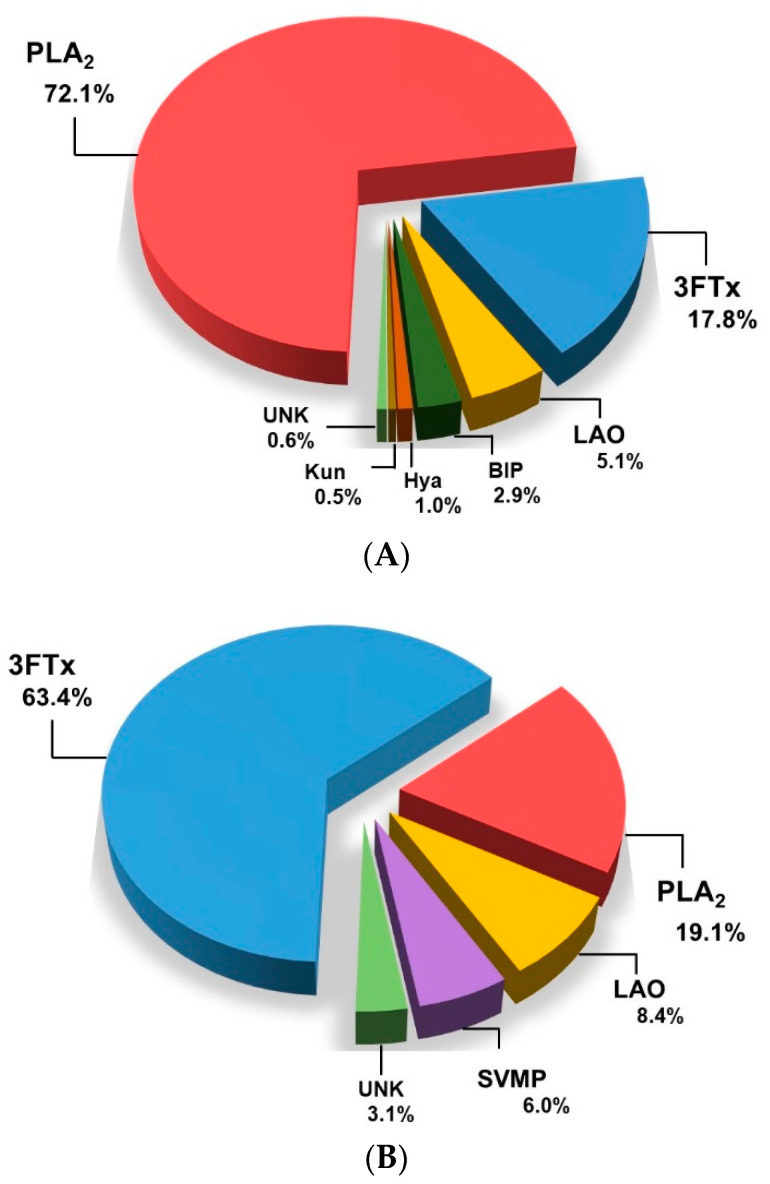
Proteomic insights into two Ecuadorian coral snake venoms. The pie charts represent the relative abundance of protein families found in the venom proteome of (**A**) *M. helleri* and (**B**) *M. mipartitus*. The relative abundance of protein families is expressed as % of the total venom toxin content, estimated based on chromatographic peak areas. UNK: unknown/unidentified.

Overall, the proteomic profiles of these two Ecuadorian coral snakes retain the hallmark proteins of *Micrurus* venom composition, where PLA_2_s or 3FTxs are the major toxins. In both venoms, the sum of the abundance of these two protein families represented more than 80% of the total proteome. Our comparative analysis exposed a 3FTx/PLA_2_ venom dichotomy in the country (Figure 5). *M. helleri* is a typical PLA_2_-predominant venom, with the PLA_2_ isoforms corresponding to 72.1% of the sample composition (Figure 5A). Conversely, the dominating biomolecules in *M. mipartitus* are 3FTxs, which constitute 63.4% of the venom proteome (Figure 5B).

## 3. Discussion

*Micrurus* (coral snake) species belong to the New World elapids, a monophyletic group of small, coloured, extremely venomous, and broadly-occurring snakes [34]. They are considered ideal organisms for ecological, evolutionary, pharmacological, physiological, phylogenetic, and proteomic studies [29]. Their ability to produce small quantities of venoms has limited the manufacturing of antivenoms as well as structural, functional, and immunological research [35]. The first integrative venom proteomics using coral snake was developed in 2008 [36], but most of the quantitative estimation of proteome compositions were published in the last 10 years [27,30]. Even so, a significant number of *Micrurus* venoms have remained inaccessible to studies [29].

The boom of proteomic approaches has led to a greater understanding of the biochemical arsenal of venoms from New World coral snakes [29,30,37]. The analysis of the distribution and abundance of toxin families has revealed two intriguing divergent compositional patterns: PLA_2_-rich and 3FTxs rich venoms [24]. In brief, a general trend marked by the polarization of venom phenotypes along *Micrurus* north–south dispersal has been observed [30]. The abundance of PLA_2_ is extremely high in venoms from coral snakes inhabiting the North, while in most southern species, this value is significantly reduced and compensated by a high expression of 3FTxs [25]. So far, proteomic analyses of coral venoms are still limited, and the challenging 3FTx/PLA_2_ dichotomy puzzle in *Micrurus* needs further pieces of information to complete a comprehensive picture.

In Ecuador, the *Micrurus* genus includes 17 species, but their venoms have not been investigated. The country does not produce antivenom and depends on importing these life-saving immunological agents [19,31]. Bothropic and Lachesis bites are covered by the polyvalent antivenom imported from Instituto Clodomiro Picado (Costa Rica) with demonstrated effectiveness, despite the high number of vials needed in some cases [31,38]. However, envenomation by elapids remains a latent problem, despite the reduced number of cases. Most public hospitals lack antivenoms against elapid snakes. To our knowledge, there are no proteomic studies of Ecuadorian coral venoms, and the preclinical efficacy of antivenoms has not been evaluated. Altogether, these antecedents motivated the present proteomic characterization of venoms from Ecuadorian coral snakes.

The recognised venom variability of *Micrurus* species earlier described [29,39] was confirmed by the comparison of the chromatograms in our study. *M. helleri* venom separation showed prominent peaks in the central chromatogram region, a higher number of fractions, and a trend of PLA_2_-rich coral venoms observed in samples from the North [40]. In contrast, the HPLC separation using the C18 column of *M. mipartitus* venom evidenced that the toxins eluted mainly in the first fractions, similar to the 3FTxs-predominant venoms largely reported from venoms from South America [41].

Electrophoresis patterns are similar to the molecular signatures of SDS-PAGE gels of coral snake venoms previously studied, such as *Micrurus clarki* [42] and *Micrurus nigrocinctus* [39]. In contrast to the wider molecular weight range of proteins found in viperid snake venoms, the elapid venom samples here evaluated presented a large content of components migrating within the range of 5–17 KDa. These results agree with the typical molecular weight of the two most abundant biomolecules (PLA_2_ and 3FTx) expressed in New World snake venoms [26,36].

Tandem mass spectrometry analysis of the tryptic-digested peptides of RP-HPLC fractions and/or gel bands against a database of venom toxins from *Micrurus species* revealed the existence of the two divergent patterns. Supporting the chromatographic and electrophoretic profiles, our findings demonstrated that *M. helleri* is a PLA_2_-predominant venom, while *M. mipartitus* exhibits higher content of 3FTxs. Therefore, in Ecuador, coral snake species with both proteomic profiles coexist, similar to Costa Rica [24], Colombia [43], and Brazil [28]. Previous studies have highlighted the implications of this venomics divergence in terms of the low cross-reactivity of elapid antivenoms across the two divergent phenotypes [20,44,45]. This fact draws attention to the need to guarantee the availability of antivenoms with wide taxonomic coverage, capable of neutralizing both venom phenotypes present in Ecuador.

*Micrurus lemniscatus* is a complex group of snakes with widely dispersed species *(M. carvalloi*, *M. helleri*, *M. diutius*, and *M. frontifasciatus*) and controversial taxonomy [46]. Different authors consider them distinct species or subspecies of *M. lemniscatus*. Some species can be also found in Colombia and Brazil [47]. The distribution of toxins in venoms from these countries was assessed in earlier studies [28], which unveiled some interesting aspects. According to the literature, the venomics profile from samples of *M. lemniscatus carvalhoi* (Brazil) showed 76.7 and 71.3% of 3FTxs [28], while *M. helleri* from Colombia has a PLA_2_-predominant phenotype, with 62.5% of the total proteome [28]. Our findings concur with the reported predominance of PLA_2_s in the venom of this subspecies, and indeed indicate higher levels of PLA_2_ expression in the Ecuadorian venom of *M. helleri* (72.1%) compared to the venom proteome of *M. helleri* from Leticia (Colombia) [28].

The 3FTx predominance characterised in *M. mipartitus* from Ecuador (63.4% of venom composition) was also reported in a previous study of Colombian and Costa Rican samples [26]. These venoms were rich in 3FTxs: 61.1% and 83%, respectively. PLA_2_ family is the second dominant toxin group found in the proteomic analysis of snake venoms from these three countries. These proteins are present in moderate proportions (19.1% of total venom toxins) in Ecuadorian *M. mipartitus*, while in samples from Colombia and Costa Rica, they account for 29.0% and 8.2% of venom proteome, respectively [26]. Additionally, Ecuadorian venom showed a higher relative abundance of SVMPs (6.0%) and LAOs (8.4%), in contrast to Colombian and Costa Rican venoms, which have a lower proportion of these catalytically active biomolecules [26]. The functional role of these toxins in the symptomatology of elapid envenomation remains unclear. The intriguing expression of haemorrhagic agents in coral snakes was also questioned in a transcriptomic study of *M. fulvius* [48]. Biological screening of the venom and clinical data reports are crucial for a better understanding of the contributions of these molecules to coral snakebites.

The pathogenesis induced by *Micrurus* venoms is characterised by neuromuscular paralysis [41]. Ecuadorian *Micrurus* snakebites have received little attention in the literature. To date, only one case report detailing the main manifestations following a *M. helleri* envenomation in Ecuador has been documented [33]. Neurotoxic effects and a mild increase in the levels of serological muscle damage biomarker (creatine kinase) were observed. These signs are probably associated with the action of PLA_2_s and 3FTxs, which account for approximately 90% of the total proteins of *M. helleri* venom. On other hand, we could not find published case reports of envenoming by Ecuadorian *M. mipartitus*. A case presentation from Colombia evidenced high neurotoxicity with progressive bilateral ptosis and respiratory paralysis [49]. The mechanism behind this characteristic clinical pattern involves the biological properties of pre-synaptically acting PLA_2_s and post-synaptic 3FTxs [29].

Similar to other investigations focused on *Micrurus* venoms, this study has some limitations. It represents an initial snapshot, since venoms were sampled from just one snake of each species, which precludes a representative picture of the total population and spatial distribution. Other earlier studies have also analysed *Micrurus* spp. venoms obtained from one specimen [26,30]. Additionally, the low venom volume obtained also hindered further functional assays and anti-venomics approaches.

In summary, new proteomic pieces were added to the current state-of-the-art information on the dichotomy of New World coral snake venoms. This study constitutes an important step toward characterizing the neglected Ecuadorian venoms and motivates further investigations with the other 15 species not yet assessed. Both contrasting venom phenotypes were evidenced in the country. *M. helleri* is a member of the group of PLA_2_-predominant types, and *M. mipartitus* is a clear example of a 3FTx-abundant venom phenotype. This finding represents the tip of the iceberg that needs to be uncovered in combination with anti-venomics approaches and preclinical evaluation of therapeutic strategies.

## 4. Materials and Methods

### 4.1. Venoms

The *Micrurus* venom samples were collected under the research permits MAE-DNB-CM-2015-0017 and MAE-DNB-CM-2019-0115 issued by the Ministry of the Environment of Ecuador. Venom was manually milked from one specimen for each species of snake: *M. helleri* and *M. mipartitus*. In this manuscript, we named *M. helleri* while taking into account the recent findings published by Hurtado-Gomes [46]. The geographical locations of individuals are detailed in Figure 2. Both animals were female adults. Venoms were dried inside a vacuum containing anhydrous calcium sulfate and stored at −20 °C until use.

### 4.2. Venom Fractionation by RP-HPLC

Decomplexation of Ecuadorian coral venoms was performed by RP-HPLC using a C18 according to a routine protocol used for the characterization of venoms from this genus [23,42]. We employed an HPLC equipped with a binary pump system (Waters 1525, Watertown, MA, USA), autosampler (Waters 2707, Watertown, MA, USA) and photodiode array detector (PDA) (Waters 991, Watertown, MA, USA). In the first step, 100–200 µg of venom samples were dissolved in 200 µL of 0.05% trifluoroacetic acid and 5% acetonitrile (*v*:*v*) in water. Then, each venom was centrifuged at 13,000× *g* for 10 min, and the supernatant was injected into a Phenomenex Jupiter C18 stationary phase 250 × 4.6 mm; 5 µm; 30 Å). Mobile phases were prepared as follows: solution A, 0.1% TFA (*v*/*v*) in distilled water; solution B, 0.1% TFA (*v*/*v*) in acetonitrile. The separation was performed at a constant flow rate of 1 mL/min for 100 min using a linear gradient: 5% of B solution, followed by 5–25% B, 25–45% B, 45–70% B, 70% B, and 45–5% B for 5, 10, 60, 10, 5, and 10 min. Fractions were manually collected based on the absorbance values, which were monitored at an absorbance of 215 nm. The relative abundance of purified molecules was quantified by integration of the peak areas using Empower software. Venom fractions were dried in a vacuum concentrator (Genevac, miVac Duo) for 24 h.

### 4.3. SDS-PAGE

RP-HPLC fractions were submitted to SDS-PAGE performed in a BIO-RAD Mini-PROTEAN Tetra with some modifications [19]. Dried fractions were dissolved in 45 µL of loading buffer (0.075 M Tris-HCl (pH 6.8); 10% (*v*/*v*) glycerol; 4% (*m*/*v*) SDS; 0.001% (*m*/*v*) bromophenol blue). The analysis was carried out under reducing conditions. Five µL of 1 M DL-dithiothreitol was added to the samples, which were heated to 95 °C in a thermoblock (Thermo Scientific, Waltham, MA, USA) for 5 min. Running buffer was prepared using (0.025 M Tris-HCl (pH 8.3); 0.192 M glycine; 0.1% (*m*/*v*) SDS). Twenty µL of samples were loaded onto a 5% stacking gel and 12.5% running gel. Five μL of a protein ladder (RunBlue Prestained TriColour) was also loaded onto the first well to estimate the molecular weight size of venom proteins. The electrophoretic separation started at 35 mA for 30 min, ending at 100 mA.

### 4.4. LC-MS/MS Analyses

Fractions obtained by RP-HPLC and protein bands visualised by SDS-PAGE were subjected to proteolytic digestion in similar manner as the protocol described by Sanz et al. (2019) [25]. The solutions were freshly prepared. In the case of electrophoresis gel, bands of interest were excised. Coomassie blue dye was extracted with 100 mM ammonium bicarbonate solution (AMBIC) and ACN (1:1) for 1 h. Proteins were reduced with DTT at 56 °C for 30 min. Alkylation was then carried out with iodoacetamide (IAA), in the dark for 20 min and followed by proteolysis with sequencing grade trypsin. AMBIC was added and incubated at 37 °C overnight. After this period, the digested peptides were extracted. The supernatant was concentrated in a vacuum centrifuge for approximately 40 min until almost dry. Finally, it was redissolved in 0.1% formic acid and stored at 4–8 °C until injection into the LC-ESI-QTOF system. The same procedure was carried out for RP-HPLC fractions when the toxin was not visible in the Coomassie blue-stained gel.

### 4.5. MS Data Processing

The resulting tryptic peptides were analysed using an ACQUITY^®^ Xevo G2-S QTof UPLC^®^/MS system fitted with the LockSpray. Mobile phases A and B consisted of 0.1% formic acid in water and 0.1% formic acid in ACN, respectively. The flow rate was set to 0.3 mL/min. The mass spectrometer operated with a capillary voltage of 0.5 kV, cone voltage, of 40 V; 120 °C source temperature; 450 °C desolvation temperature; 30 L/h cone gas flow; and 900 L/h of desolvation gas flow.

The LC gradient went from 1% to 70% B in 30 min. MS data were acquired using the data-dependent acquisition (DDA) method using a mass scan in the m/z region from 400 to 1990 Da. The most abundant ions in the MS1 spectra with charges +2 and +3 were selected for fragmentation (MS2) by collision-induced dissociation (CID). Fragment spectra were interpreted using a licensed version of the MASCOT DISTILLER 2.7 program (https://www.matrixscience.com/distiller.html) against the UniProt/SwissProt database for Serpentes. We also created a database including the primary structure of toxins from *Micrurus* spp. and other genera of the Elapidae family. The MS/MS tolerance mass was set to 0.6 Da, similar to a previous study with *Micrurus pyrrhocryptus* venom [39]. Carbamidomethyl cysteine and oxidation of methionine were defined as fixed and variable modifications, respectively.

## Figures and Tables

**Table 1:** Overview of MS/MS identification of *M. helleri* toxins found in SDS-PAGE gel and/or RP-HPLC fractions. Protein assignment was achieved by matching MS/MS-derived peptide sequences with snake venom toxin databases.

Fractions	Relative Abundance	Peptide Ion		Peptide Sequences	Related Proteins		
		m/z	Z		Family	Species	Database
1	0.65	Unknown/unidentified					
2	3.79	414.80	3+	TRCDGFCGNR	3FTx	*Naja atra*	E2IU01
		461.23	2+	TIDECQR	SP Kunitz inhibitor	*Bungarus fasciatus*	P25660
		532.28	2+	TPPAGPDVGPR	Bradykinin inhibitor peptide	*Agkistrodon bilineatus*	P85025
3	0.78	414.80	3+	TRCDGFCGNR	3FTx	*Naja atra*	E2IU01
4	5.34	414.80	3+	TRCDGFCGNR	3FTx	*Naja atra*	E2IU01
5	1.83	520.73	2+	YNKISFIR	3FTx	*Micrurus altirostris*	F5CPD4
6	2.42	532.28	2+	TPPAGPDVGPR	Bradykinin inhibitor peptide	*Agkistrodon bilineatus*	P85025
7	0.46	484.27	2+	IICCRSC	3FTx	*Micrurus frontralis*	P86420
8	0.40	484.27	2+	IICCRSC	3FTx	*Micrurus frontralis*	P86420
9	0.51	484.27	2+	IICCRSC	3FTx	*Micrurus frontralis*	P86420
10	0.16	457.33	3+	FCELPADSGSCK	SP Kunitz inhibitor	*Phesudechis rossignolii*	E7FL13
		464.69	3+	GCASSCPKNGLIK	3FTx	*Micrurus diastema*	A0A0H4BKJ5
11	5.43	464.69	3+	GCASSCPKNGLIK	3FTx	*Micrurus diastema*	A0A0H4BKJ5
12	0.94	447.24	2+	LAALCFAK	PLA_2_	*Micrurus mipartitus*	C0HKB9
13	0.41	447.24	2+	LAALCFAK	PLA_2_	*Micrurus mipartitus*	C0HKB9
14	6.88	447.24	2+	LAALCFAK	PLA_2_	*Micrurus mipartitus*	C0HKB9
15	4.94	582.32	2+	NLVQFGNMIK	PLA_2_	*Micrurus lemniscatus carvalhoi*	A0A2H6NAU5
		594.78	2+	GGSGTPVDDLDR	PLA_2_	*Micrurus spixii*	A0A2D4NMB1
16	3.34	594.78	2+	GGSGTPVDDLDR	PLA_2_	*Micrurus spixii*	A0A2D4NMB2
17	1.25	594.78	2+	GGSGTPVDDLDR	PLA_2_	*Micrurus spixii*	A0A2D4NMB3
18	33.72	594.78	2+	GGSGTPVDDLDR	PLA_2_	*Micrurus spixii*	A0A2D4NMB4
		687.26	2+	CQDFVCNCDR	PLA_2_	*Micrurus lemniscatus carvalhoi*	A0A2H6NAU5
		440.72	2+	VAANCFAK	PLA_2_	*Micrurus lemniscatus carvalhoi*	A0A2H6NAU6
		590.31	2+	NLVQFGNM^OX^IK	PLA_2_	*Micrurus lemniscatus carvalhoi*	A0A2H6NAU5
		566.27	2+	GSGTPVDDLDR	PLA_2_	*Micrurus lemniscatus carvalhoi*	A0A2H6NFP9
		503.74	2+	MIECANIR	PLA_2_	*Micrurus lemniscatus carvalhoi*	A0A2H6NFP10
		687.26	2+	CKDFVCNCDR	PLA_2_	*Micrurus dumerilii*	C0HKB8
19	17.41	594.77	2+	GGSGTPVDDLDR	PLA_2_	*Micrurus spixii*	A0A2D4NMB1
		440.73	2+	VAANCFAK	PLA_2_	*Micrurus surinamensis*	A0A2D4PZ69
20	1.04	521.21	2+	AFVCNCDR	PLA_2_	*Micrurus mipartitus*	C0HKB9
21	1.82	521.21	2+	AFVCNCDR	PLA_2_	*Micrurus mipartitus*	C0HKB9
22	0.38	521.21	2+	AFVCNCDR	PLA_2_	*Micrurus mipartitus*	C0HKB9
23	0.96	721.69	3+	ISFM^OX^TAHDYSLPVFVYTR	HYA	*Micrurus corallinus*	A0A2D4H401
24	4.22	742.85	2+	EADYEEFLEIAR	LAO	*Micrurus tener*	A0A194ARE6
		577.78	2+	FDEIVGGFDR	LAO	*Micrurus tener*	A0A194ARE7
		742.85	2+	EADYEEFLEIAR	LAO	*Micrurus mipartitus*	A0A2U8QNR2
		497.24	2+	FWEADGIR	LAO	*Micrurus mipartitus*	A0A2U8QNR3
		746.88	2+	FDEIVGGFDRLPK	LAO	*Micrurus paraensis*	A0A2D4K1Y6
		586.29	2+	DHGWIDSTIK	LAO	*Micrurus paraensis*	A0A2D4KMW9
		627.32	2+	SASQLYQESLK	LAO	*Micrurus paraensis*	A0A2D4KYN2
25	0.93	742.85	2+	EADYEEFLEIAR	LAO	*Micrurus mipartitus*	A0A2U8QNR2

Abbreviations: 3FTx: three-finger toxin; PLA_2_: phospholipase A_2_; HYA: hyaluronidase and LAO: L-amino acid oxidase.

**Table 2:** Overview of MS/MS identification of *M. mipartitus* toxins found in SDS-PAGE gel and/or RP-HPLC fractions. Protein assignment was achieved by matching MS/MS derived peptide sequences with snake venom toxin databases.

Fractions	Relative Abundance	Peptide Ion		Peptide Sequences	Related Proteins		
		m/z	z		Family	Species	Database
1	3.07	Unknown/unidentified					
2	0.30	559.28	2+	GCAVTCPKPK	3FTx	*Micrurus mipartitus*	A0A2P1BSS8
3	0.17	559.28	2+	GCAVTCPKPK	3FTx	*Micrurus mipartitus*	A0A2P1BSS8
4	2.06	733.34	2+	KGIEINCCTTDR	3FTx	*Naja sputatrix*	O57327
		676.02	3+	VDLGCAATCPKVKPGVNIK	3FTx	*Naja nivea*	P01390
		733.34	2+	KGIELNCCTTDR	3FTx	*Naja mossambica*	P01431
5	7.61	733.34	2+	KGIELNCCTTDR	3FTx	*Naja mossambica*	P01431
6	40.26	733.34	2+	KGIELNCCTTDR	3FTx	*Naja mossambica*	P01431
		722.34	2+	LVPLFSKTCPPGK	3FTx	*Naja atra*	P60307
7	1.07	733.34	2+	KGIEINCCTTDR	3FTx	*Naja sputatrix*	O57327
8	1.73	733.34	2+	KGIEINCCTTDR	3FTx	*Naja sputatrix*	O57327
9	7.89	447.25	2+	LAALCFAK	PLA_2_	*Micrurus mipartitus*	C0HKB9
		521.22	2+	AFVCNCDR	PLA_2_	*Micrurus mipartitus*	C0HKB9
10	5.54	440.78	2+	VAANCFAK	PLA_2_	*Micrurus surinamensis*	A0A2D4PZ69
		447.73	2+	VAAKCFAK	PLA_2_	*Micrurus surinamensis*	A0A2D4PRR8
11	5.16	448.73	2+	VAAKCFAK	PLA_2_	*Micrurus surinamensis*	A0A2D4PRR8
12	0.51	447.73	2+	VAAKCFAK	PLA_2_	*Micrurus surinamensis*	A0A2D4PRR8
13	3.89	841.43	3+	KTLLLNLVVVTIVCLDFGYTIK	3FTx	*Bungarus flaviceps*	D5J9P5
14	4.06	841.43	3+	KTLLLNLVVVTIVCLDFGYTIK	3FTx	*Bungarus flaviceps*	D5J9P5
15	1.26	841.43	3+	KTLLLNLVVVTIVCLDFGYTIK	3FTx	*Bungarus flaviceps*	D5J9P5
16	0.99	775.91	2+	CVINATGPFTDTVR	3FTx	*Micrurus lemniscatus lemniscatus*	A0A2D4IKM1
17	0.97	694.86	2+	YIEFYVAVDNR	SVMP	*Bungarus multicinctus*	A8QL49
18	0.72	705.36	3+	IDFNGNTLGLAHIGSLCSPK	SVMP	*Micrurus fulvius*	U3EPC7
		632.84	2+	SNVAVTLDLFGK	SVMP	*Micrurus fulvius*	U3EPC7
		708.86	2+	YIEFYVVVDNR	SVMP	*Micrurus fulvius*	U3EPC7
19	4.30	660.34	2+	KMNDNAQLLTR	SVMP	*Micrurus fulvius*	A0A0F7YYV1
		503.95	3+	RPECILNKPLNR	SVMP	*Micrurus fulvius*	A0A0F7YYV1
20	8.45	934.97	2+	TLPSVTADYVIVCSTSR	LAO	*Micrurus mipartitus*	A0A2U8QNR6
		624.37	2+	KVIVTYQTPAK	LAO	*Micrurus mipartitus*	A0A2U8QNR6
		649.02	3+	HVVVVGAGM^OX^SGLSAAYVLAK	LAO	*Micrurus mipartitus*	A0A2U8QNR6
		742.85	2+	EADYEEFLEIAR	LAO	*Micrurus mipartitus*	A0A2U8QNR6
		519.79	2+	IFLTCTKR	LAO	*Micrurus mipartitus*	A0A2U8QNR6
		568.79	2+	IHFAGEYTAK	LAO	*Micrurus mipartitus*	A0A2U8QNR6
		742.85	2+	EADYEEFLEIAR	LAO	*Micrurus tener*	A0A194ARE6
		627.32	2+	SASQLYQESLK	LAO	*Micrurus paraensis*	A0A2D4KYN2

Abbreviations: 3FTx: three-finger toxin; PLA_2_: phospholipase A_2_; LAO: L-amino acid oxidase and SVMP: snake venom metalloproteinase.

## Data Availability

The original contributions presented in this manuscript are publicly available.
